# The Role of Na/K-ATPase Signaling in Oxidative Stress Related to Aging: Implications in Obesity and Cardiovascular Disease

**DOI:** 10.3390/ijms19072139

**Published:** 2018-07-23

**Authors:** David E. Bartlett, Richard B. Miller, Scott Thiesfeldt, Hari Vishal Lakhani, Joseph I. Shapiro, Komal Sodhi

**Affiliations:** 1 Department of Internal Medicine, Joan C. Edwards School of Medicine, Marshall University, Huntington, WV 25701, USA; bartlett14@marshall.edu (D.E.B.); miller1088@marshall.edu (R.B.M.); thiesfeldt@marshall.edu (S.T.); lakhani@marshall.edu (H.V.L.); shapiroj@marshall.edu (J.I.S.); 2 Department of Surgery and Biomedical Sciences, Joan C. Edwards School of Medicine, Marshall University, Huntington, WV 25755, USA

**Keywords:** aging, obesity, cardiovascular disease, oxidative stress, Na/K-ATPase signaling

## Abstract

Aging has been associated with a series of pathophysiological processes causing general decline in the overall health of the afflicted population. The cumulative line of evidence suggests an important role of oxidative stress in the development and progression of the aging process and metabolic abnormalities, exacerbating adipocyte dysfunction, cardiovascular diseases, and associated complications at the same time. In recent years, robust have established the implication of Na/K-ATPase signaling in causing oxidative stress and alterations in cellular mechanisms, in addition to its distinct pumping function. Understanding the underlying molecular mechanisms and exploring the possible sources of pro-oxidants may allow for developing therapeutic targets in these processes and formulate novel intervention strategies for patients susceptible to aging and associated complications, such as obesity and cardiovascular disease. The attenuation of oxidative stress with targeted treatment options can improve patient outcomes and significantly reduce economic burden.

## 1. Introduction

**Figure 1:**
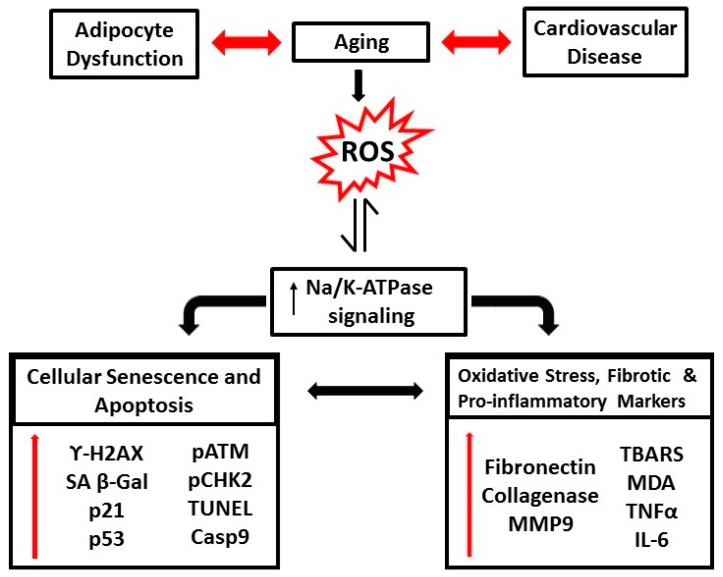
Schematic representation of the pathophysiological alterations in the biological aging process, in association with obesity and cardiovascular disease.

Aging is an incidental and irreversible process that is often augmented by alterations in the cellular mechanisms including, but not limited to, irrevocable DNA damage, impaired nuclear activity, compromised cell division, apoptotic processes, and oxidative stress [1,2,3,4,5]. This process is often characterized by phenotypic changes and an overall decline in physiological processes that further contributes to age-related complications. Numerous studies have demonstrated, yet not fully established, that aging has a causal relationship with metabolic derangements such as obesity and augment the risk of cardiovascular diseases, including atherosclerosis, hypertension, and myocardial infarction, due to increased hypertrophy, fibrosis, and an elevated number of apoptotic and necrotic cells. This association has been summarized in Figure 1.

The trigger for the age-associated complications, such as obesity and cardiovascular diseases, is intricately linked with an increase in reactive oxygen species (ROS) and subsequent oxidative stress [6,7]. The primary sources of endogenous intracellular ROS include cellular mitochondria, endoplasmic reticulum (ER) stress, activation of the Ras pathway, and upregulated activity of NADPH oxidase (NOX) [8]. Evidence suggests that ROS-induced oxidative stress is critical in aging, exacerbating cellular injury and DNA damage, resulting in senescence and apoptosis [8,9]. Oxidative stress in aging accentuates cardiac fibrosis and inflammation, further leading to overexpression of chemokines and pro-inflammatory cytokines, giving rise to cardiovascular complications and disorders [10].

While there are multiple sources of ROS in aging and associated cardiovascular disorders, recent studies have established a mechanistic role of sodium potassium adenosine triphosphatase (Na/K-ATPase) signaling cascade in the exacerbation of oxidative stress [11,12,13]. Xie and his group discovered the scaffolding and signaling function of Na/K-ATPase, apart from its pumping function, over two decades ago. Since then, our group has demonstrated multiple lines of evidence suggesting the involvement of Na/K-ATPase signaling in various disease models ranging from obesity to cancer, including our recently published data in aging models [14,15,16]. These studies target Na/K-ATPase signaling cascade with the goal of developing therapeutic options for the clinical disorders, which are all associated to systemic and cellular redox imbalance [12,17,18]. Thus, understanding the role of Na/K-ATPase signaling in oxidative stress related pathophysiology of aging and subsequent cardiovascular disease is important.

## 2. Na/K-ATPase Signaling and Oxidative Stress

**Figure 2:**
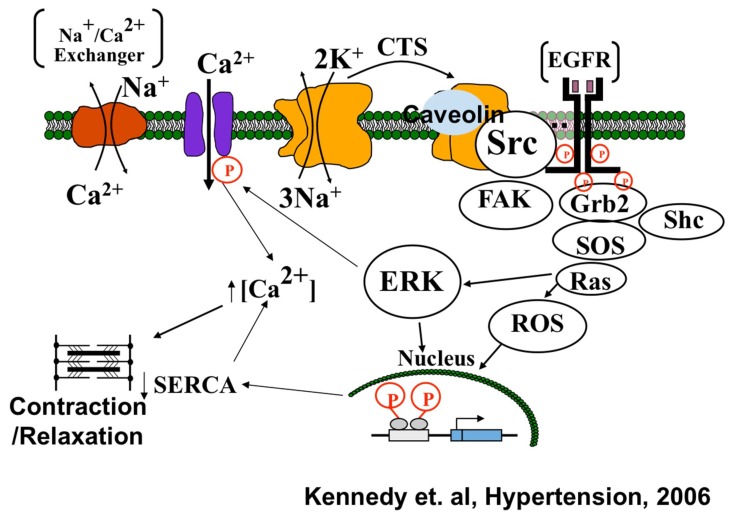
Xie model for Na/K-ATPase signaling showing Na/K-ATPase (orange proteins) serving as scaffolding protein in caveolae regulating the tyrosine kinase activity of Src. Na/K-ATPase can act as a specific receptor for CTS and as a non-specific receptor for ROS. As this signal cascade generates ROS, the Na/K-ATPase-Src cascade can serve as a feed forward amplifier for ROS. Source: Kennedy et al. (2006) [26].

The Na/K-ATPase, a P-type ATPase, has recently been shown by Xie/Shapiro laboratories to cause oxidative stress through mechanisms distinct from its well-understood pumping function (Figure 2) [19,20,21]. The major thrust of the Xie/Shapiro laboratories over the past twenty years has been the elucidation of a signaling function for the Na/K-ATPase that is based on the scaffolding properties of the α1 subunit. The Na/K-ATPase α-1 subunit causes structural modulation leading to the activation of membrane bound Src kinase [13,21]. The carbonylation of α-1 subunit is induced by excessive systemic ROS, which is greatly produced in a diseased condition, leading to the formation of α-1/Src complex, resulting in phosphorylation and activation of Src [13,22]. The activation of Src kinase further leads to the downstream activation of signaling cascade including Ras-Raf-MEK-ERK pathway resulting in the production of excessive mitochondrial ROS. Hence, it has been evidently demonstrated that the production of ROS is not just a consequence of Na/K-ATPase signaling activation, but also plays a role in its initiation, therefore creating a Na/K-ATPase oxidant amplification loop [23,24]. Evidence suggests that the activation of Src also regulates NADPH oxidase-derived superoxide generation [25]. Protein carbonylation has also been well established as a marker for the modulation of the Na/K-ATPase signaling function under oxidative stress. It has also been demonstrated that cardiotonic steroids (CTS) and glucose-oxidase-induced H_2_O_2_ trigger the direct carbonylation of the α-1 subunit, which promotes a feed forward mechanism of Na/K-ATPase signal transduction [22]. Since oxidative stress plays a key role in the progression of aging process and neurodegenerative diseases, it is imperative to examine potential targets that can restore redox imbalance.

## 3. The Development of pNaKtide

**Figure 3:**
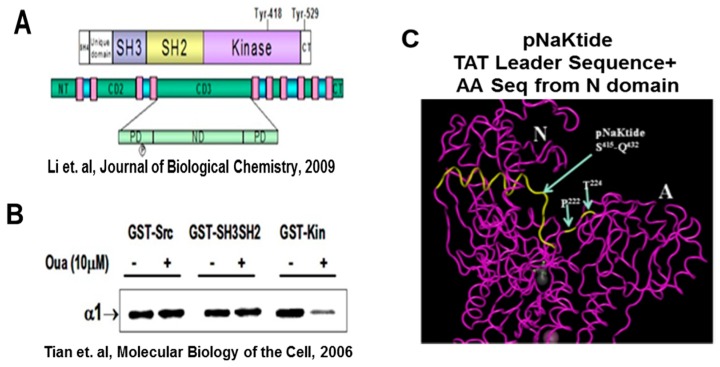
Depiction of alignment of the Na/K-ATPase alpha1 subunit with the Src Kinase domain. Further exploration defined a 20-aminoacid sequence in the N domain (part of CD3 domain) of alpha1, which binds Src’s kinase domain (NaKtide). (**A**) Schematic illustration of the structure of Src representing the SH3-SH2 domain, tyrosine kinase, and different GST-fusion proteins [27]. (**B**) Western blot analysis demonstrating the ouabain-induced release of kinase domain from the Na/K-ATPase, blotted against anti-α-1 antibody [28]. (**C**) Schematic structural representation of a portion of the TAT protein to form a 33 AA cell permeant peptide, pNaKtide. Source: Tian et al. (2006) and Li et al. (2009) [27,28].

In concordance to the previous observations and the critical role of Na/K-ATPase signaling in clinical disorders, Xie’s group developed a peptide, pNaKtide that specifically acts as an antagonist of the scaffolding function of Na/K-ATPase signaling. The specific details for the development of pNaKtide have been outlined in 2009 [27]. Briefly, the portion of the α-1 subunit that interacts with the Src kinase domain was used in this sequence to develop an effective peptide, which will allow for the inhibition of Src kinase. This peptide was further merged with the HIV-Tat (TAT) leader sequence to facilitate cellular permeation (Figure 3). The exclusivity of this peptide was noted by demonstrating its localization to the membrane component of the cell that essentially impede its inhibition to membrane associated Src. Consistently, this peptide, pNaKtide, hindered the formation of the Na/K-ATPase-Src complex, hence inhibiting the activation of further downstream signaling cascade. Interestingly, pNaKtide did not alter the highly regulated Src activity and did not impair the unique ionic pumping function of Na/K-ATPase.

Multiple studies have demonstrated the effectiveness of pNaKtide and compared it with similar Src inhibitors, such as PP2. However, unlike other Src inhibitors, pNaKtide offers specificity to the Na/K-ATPase complex and evidently blocks the formation of the Na/K-ATPase/Src receptor complex, while inhibiting ouabain-induced activation of ERK1/2 and hypertrophic growth in cardiac myocytes [27]. Hence, pNaKtide functions in a specific manner and has been found to reduce oxidative stress and inhibit ROS-induced Src activation in cell preparations as well as in animal models of disease. Antioxidants alone have thus far failed in pre-clinical and clinical trials, which could be related to the lack of “range” related to specific therapy; once the scavengers are consumed, the underlying process continues as before. This peptide has been demonstrated to effectively attenuate the disease progression in the in vitro and in vivo models of studies by mediating the blockage of the Na/K-ATPase/ROS amplification loop, conclusively providing a strong evidence of its therapeutic efficacy, as well as furthering our understanding concerning the basic, cellular mechanisms operant in several clinical disorders and aging.

The cellular generation of ROS has been demonstrated to be attenuated by the administration of pNaKtide. In vitro and in vivo studies have provided significant evidence suggesting a strong potency of pNaKtide in ameliorating multiple clinical disorders including, but not limited to, experimental uremic cardiomyopathy, steatohepatitis, and atherosclerosis [12,18]. The recent studies effectively characterize the therapeutic potential of pNaKtide, by antagonizing Na/K-ATPase-mediated amplification of ROS signaling, subsequently preventing or reversing the diseased phenotype and restoring metabolic homeostasis in a dose-dependent manner, in murine models [11]. The ongoing research and future studies, on the effectiveness of pNaKtide, will potentially bridge the gap between the rodents and the humans by demonstrating its safe administration, further allowing the clinical use of pNaKtide and possibly reversing the clinical disorders. 

## 4. The Role of Oxidative Stress and Na/K-ATPase/ROS Signaling in Aging

Aging is a complex, multifactorial process by which an organism, at the genetic, cellular, and organ-system level deteriorates. At the molecular level, aging has been associated with an increase in DNA mutations, telomere shortening, and changes in methylation patterns, each of which can disturb the normal expression and/or function of proteins involved in cell growth, genomic integrity, cellular stress responses, and inflammation [29,30]. Reflecting on U.S. Census population statistics and predictions, people aged 65 and older represented 15% of the total U.S. population in 2016. The future projections for the aging population suggest a rise to 22% of the population by 2050. In addition, the aged population over 85 years contributed towards 2% of the total U.S. population in 2016 with an expected rise to 5% by 2050. Given these trends, it is imperative to understand the molecular mechanisms critical to aging process and its associated complications.

Though the underlying mechanisms of aging remain poorly understood, a growing body of evidence points towards ROS as the causative agents of DNA, lipid, and protein damage that are associated with impairment at the cellular and tissue level [31,32,33,34]. When ROS accumulation exceeds the detoxifying ability of the cell, the resulting oxidative stress can induce damage, such as increased DNA mutations and destabilization of electron transport chain complexes, and can cause senescence or eventual apoptosis [35,36,37]. Given the complexity of the aging process, one of the most widely known concept is the “free radical theory of aging,” which was initially coined in 1956 [38,39]. This theory extensively explains the critical role of ROS as a major determinant of lifespan while driving multiple aging processes [8]. This theory further elaborates the role mitochondrial processes in the generation of ROS, causing mtDNA point mutations, damage to cellular macromolecules, and alterations in the NADPH equilibrium. Intracellular ROS has been evidently reported to be directly and indirectly involved in the telomere shortening, which is a major cause of replicative senescence and a common process of aging. This is often associated with the significant reduction of superoxide dismutase (SOD) and the loss of ROS scavengers such as N-acetyl cysteine (NAC), which inhibits the ability of the cell to delay the onset of cellular senescence [40]. Cell senescence is largely induced and maintained through the p53 pathway [29,41,42]. In the p53 pathway, the effects of oxidative stress, such as double-strand breaks in DNA, trigger activation of ATM and CHK2, which activates p53 [29,35,43,44]. Activated p53 upregulates p21, which in turn inhibits CDK2 from phosphorylating Rb and arrests cells at the G_1_/S checkpoint [35,43,44,45]. Collectively, mitochondrial dysfunction and damage, cellular senescence, apoptosis, and altered cellular homeostasis activates the inflammatory pathways, engaging the immune system into a pro-inflammatory state, upregulating associated chemokines and cytokines, including, but not limited to, thromboxane A2, IL-1β, TNF-α, and IL-6 [46,47]. Such age-related systemic inflammation has been termed as “inflammaging,” exacerbating the aging process [48]. Understanding the interactions between these factors and redox imbalance is vital in providing a deeper insight in the aging process and associated complications.

Since oxidative stress plays a key role in the process of aging, our group examined a potential therapeutic target that can restore the oxidant equilibrium. We demonstrated the role of the Na/K-ATPase oxidant amplification loop in the exacerbation of oxidant injury in aging, while the peptide, pNaKtide, negated the activation of the Na/K-ATPase signaling cascade. We established an in vivo model of aging, using 2-month-old and 16-month-old C57BL/6 mice. The results showed an increase in body weight, visceral fat, and subcutaneous fat in old mice, while consistent with these observations, the locomotor activity and the overall energy expenditure was shown to be decreased, as compared to the young mice. These differences were negated by the administration of pNaKtide in old mice, suggesting a strong efficacy of this peptide. Because senescence is a hallmark of aging, we investigated if modulating Na/K-ATPase signaling affected oxidation-induced cell senescence in vitro. We performed in vitro studies in human dermal fibroblasts (HDFs), inducing senescent phenotype by treatment with H_2_O_2_, ouabain (CTS), ultraviolet radiation, and glucose oxidase (GO). We demonstrated the overexpression of oxidation-induced senescent and apoptotic markers, including p21, p53, pATM, pCHK2, ApoJ, fibronectin, MMP9, collagenase, γ-H2AX, and SA β-Gal, which was ameliorated by the Na/K-ATPase signaling antagonist, pNaKtide. In addition to senescence, cell injury and apoptosis are all markers of oxidative stress [49]. Evidence suggests that, during the aging process, there is a significant release of H_2_O_2_ by mitochondria that aids in inducing oxidative stress. In our study, H_2_O_2_ and ouabain exposure increased levels of cell injury and apoptotic markers. Subsequent pNaKtide treatment decreased these factors, supporting the hypothesis that Na/K-ATPase signaling affects the overall oxidation levels in cells. To ensure that the above effects were the result of Na/K-ATPase signaling, we looked at markers of signaling activation: protein carbonylation and phosphorylation of Src. Previous studies have shown that α1 carbonylation is a critical mediator of Na/K-ATPase signaling under oxidative stress conditions. Consistent with previous studies, protein carbonylation and Src phosphorylation increased in our in vitro and in vivo aging models and decreased following pNaKtide treatment. Taken together, these data indicate that inhibition of Na/K-ATPase signaling was able to reverse oxidation-induced senescence and apoptosis at both the morphological and molecular level [16].

## 5. The Role of Oxidative Stress and Na/K-ATPase/ROS Signaling in Aging and Associated Obesity

Obesity is regarded as an excess accumulation of adipose tissue, resulting in a body mass index (BMI) greater than 30. Obesity and aging has been implicated in multiple studies, suggesting the aggravation of the biological aging process. The prevalence of obesity in the population aged 60 years and older was 41.0% in the United States between 2015 and 2016 [50]. The risk ratio of all-cause mortality for people who were obese and were 50 years of age and older was 1.25, highlighting the importance for investigating the underlying mechanisms which contribute to both age and obesity [51]. This could potentially alleviate many burdens imposed on both patients and the healthcare system.

Oxidative stress is a key inducer of obesity through induction of adipogenesis and dysregulation of adipocyte phenotype [52]. This oxidative-stress-induced phenotypic alteration increases adipogenic markers such as peroxisome proliferation factor-γ (PPAR-γ) [53], fatty acid synthase (Fas) [54], and mesoderm-specific transcript protein (Mest) [55], along with pro-inflammatory cytokines including TNF-α and IL-6, which exacerbates the aging process [56], causing senescence and DNA damage. The production of ROS characterized in obesity and aging, which leads to oxidative stress, can be partly attributed to mitochondrial dysfunction, which is further exacerbated by decreased levels of antioxidants [57,58]. The interplay between obesity and oxidative stress in adipose tissue modifies the secretome toward a pro-inflammatory composition of adipokines [59,60]. This altered secretome generates low grade (chronic) inflammation, which exacerbates adipocyte dysfunction and impairs the metabolic profile to increase levels of lipids, free fatty acids (FFAs), glucose, and ROS when aging and obesity coexist [61]. This process creates a vicious cycle of ROS production and the release of pro-inflammatory cytokines in obesity and aging [59]. Oxidative stress and low grade (chronic) inflammation observed in obesity accelerates the shortening of telomeres by damaging G-rich sequences, which is further intensified by a reduced capacity of DNA repair in these sequences, suggesting a hastened aging process [62]. To further strengthen the evidence that obesity exacerbates aging, various studies in humans have demonstrated an inverse correlation between telomere length and abdominal adiposity [63], as well as p53 protein pathway, a key regulatory pathway in aging, playing a vital role in adipose tissue associated with obesity [64]. Aging and obesity synergistically lead to the accumulation of senescent cells along with senescence-associated secretory phenotype (SASP) generating further increased DNA damage and oxidative stress [65]. Aging and obesity reduce longevity by deleterious effects on the structure and function of organs due to oxidative stress, genetic instability, and disturbances of homeostatic pathways [66]. The demonstration that the Na/K-ATPase oxidant amplification loop plays a key role in the generation and amplification of ROS highlights the importance of investigating its role in diseases such as obesity that are intimately linked to high levels of oxidative stress [13,22]. The Na/K-ATPase oxidant amplification loop has been shown to induce adipocyte dysfunction in murine preadipocyte (3T3-L1) cell lines, and pNaKtide attenuated not only the oxidant stress but also adipogenesis [11]. A recent study showed that 16-month-old C57BL/6 mice fed a high fat diet significantly increased body weight, visceral fat, and subcutaneous fat, as compared to young and old mice fed a normal chow diet. These levels were attenuated after the administration of pNaKtide [16]. This exemplifies the importance of utilizing Na/K-ATPase signaling as a therapeutic target in an effort to ameliorate aging and obesity.

## 6. The Role of Oxidative Stress and Na/K-ATPase/ROS Signaling in Aging and Associated Cardiovascular Diseases (CVDs)

Age is a major risk factor for cardiovascular diseases (CVDs), not only because it prolongs exposure to several other cardiovascular risks but also because of its intrinsic cardiac aging. Intrinsic cardiac aging is defined as the slowly progressive age-dependent degeneration and decline in function, which makes the heart more vulnerable to stress and contributes to increased cardiovascular morbidity and mortality in the elderly. Almost 85.6 million American adults are projected to have one or more type of CVD, while 43.7 million of them are estimated to be greater than or equal to 60 years old [67]. Approximately two-thirds of CVD mortality occurs in people 75 years of age or older, making it a principle cause of death globally. The socioeconomic burden for CVD patients 65 years and older was estimated to be $116.9 billion [67]. The mechanisms operant in this frightening clinical phenotype is unclear but appears to involve the constellation of severe diastolic dysfunction, left ventricular hypertrophy, and systemic oxidant stress [17,68]. A number of investigators have proposed that oxidant stress is both a feature of this clinical syndrome as well as a pathophysiological contributor or cause [69,70,71].

Oxidative stress plays a central role in the biological aging process, resulting in the decline of cellular defensive mechanisms that scavenges ROS. The cardiovascular system in the aging process is more prone to damage by the excessive intracellular ROS due to the presence of a significantly higher mitochondrial activity, which is a main source of ROS generation, in the myocardium. A substantial line of evidence suggests that excessive ROS in the myocardium can cause myocardium senescence, consequently leading to apoptosis, endoplasmic reticulum (ER) stress, lipofuscin formation, and activation of pro-inflammatory pathways [7,38,72]. Age-induced oxidative stress is paramount in contributing towards cardiovascular and cardiac structural damage. With aging, the endothelial cells and vascular smooth muscle cells produce ROS, which interacts with nitrogen species, further oxidizing the low-density lipoproteins (LDL) that migrate to cardiac vasculature, exacerbating atherosclerosis. Numerous studies have also demonstrated a strong correlation between the age-related excess ROS generation and an inclined cardiac myocyte death [38]. The various well-studied sources of ROS generation in CVD includes impaired NADPH oxidase (NOX) activity, mitochondrial dysfunction, xanthine oxidase, and uncoupled nitric oxide synthases [73]. Consistent with the noted increase in the endogenous ROS production, there is significant reduction of greater than 50% in antioxidant enzyme activity, including manganese superoxide dismutase (MnSOD), catalase (CAT), glutathione peroxidase (GPx), and glutathione *S*-transferase (GST), as noted in the cardiovascular system of rodents [74]. Additionally, cardiac aging in the murine model demonstrated a significantly greater lipid peroxidation in the aged heart when compared to the heart tissues from young mice [74]. Such decline in heart function due to the age-related oxidative stress often leads to a significant reduction in cardiac output and heart rate, which encapsulates structural changes such as fibrosis [7].

Cardiac aging in the murine model closely recapitulates the age-related cardiac changes, which has been demonstrated by our recent study that establishes a basis for the role of the Na/K-ATPase oxidant amplification loop in exacerbating the cardiac damage. Transthoracic Echocardiography performed on an in vivo aging model demonstrated that there were significant age-dependent linear trends in increased left-ventricular mass index (LVMI), posterior wall thickness (PWT) and relative wall thickness (RWT), in the oldest group compared to young controls. The myocardial performance index significantly worsened with age and indicates that a greater fraction of systole is spent to cope with the pressure changes during isovolumic phases. However, administration of pNaKtide attenuated these impairments and improved cardiac function. Further evidence demonstrated a significant increase in senescence and apoptotic markers, including ApoJ, fibronectin, and p21, in the heart tissue of C57BL/6 aging mice, which was negated by pNaKtide. The determination of magnitude of cardiac fibrosis, in the heart tissue of aging mice, was shown to be excessive with a clear demonstration of fragmented degraded myofibers. However, the Na/K-ATPase antagonism through pNaKtide attenuated this cardiac damage. Protein carbonylation and Src phosphorylation, in vivo, suggests the importance of the Na/K-ATPase oxidant amplification loop in aggravating the detrimental cardiovascular damage in aging [16].

## 7. Pharmacological Interventions for Aging

Extensive research on the manifestation of biological aging process has presented with various pharmacological interventions that can improve longevity and battle the underlying mechanisms operant in age-associated cellular senescence and apoptosis. One of the intervention strategy involves the modulation of senescent associated secretory phenotype (SASP) regulatory pathways that acts by the interference with paracrine signaling of the senescent cells. The key factor involved in the SASP regulation includes the stimulation of the NF-κB pathway [75,76], the mammalian target of rapamycin (mTOR) pathway [77,78], the mitogen activated protein kinase (MAPK) [79], and the activation of pro-inflammatory pathways. Another important marker associated with SASP regulation is transcription growth factor-β (TGFβ), which has the potential to induce senescence via the autocrine and paracrine signaling, allowing for the release of excessive ROS in the system, triggering DNA damage and causing cell cycle arrest [80,81]. In concordance to the activation of these pathways, some commercially available therapeutic interventions include Rapamycin, which inhibits the mTOR pathway, Resveratrol and Metformin, which inhibit the NF-κB pathway, and Cortisol/Corticosterone, which is a group of steroid hormones inhibiting the release of pro-inflammatory cytokines [82,83]. Some anti-aging therapies involve the selective elimination of senescent cells by the modulation of pathways that contribute to apoptosis, including the PI3K and Bcl-2 pathways. These pathways are impeded by the use of recently classified senolytic drugs, including quercetin, dasatinib, and navitoclax [82,84,85].

Although these drugs have demonstrated a great pharmacological impact on aging, studies have shown less selectivity of these drugs, towards underlying pathways [82]. Based on these observations, our peptide, pNaKtide, has shown specific antagonism to the Na/K-ATPase signaling pathway, which plays a critical role in the amplification of oxidative stress in several clinical disorders including aging. We have established the critical role of the Na/K-ATPase oxidant amplification loop, which has allowed for the exploration of developing a new pharmaceutical approach to improving longevity and diseased physiology.

## 8. Conclusions

This review aims to highlight the significance of the Na/K-ATPase oxidant amplification loop in the process of aging as well as associated obesity and cardiovascular disease. We demonstrate that the aging process propagates cellular oxidative stress, senescence, and apoptosis, activating inflammatory pathways and cellular signaling pathways, such as Na/K-ATPase, which exacerbates the pathophysiological condition. Aging-induced oxidative stress also leads to alterations of adipocyte phenotype and dysfunction in an obese state, while causing cardiac vasculature damage and fibrosis, further amplifying cardiovascular diseases. The recently established role of activated Na/K-ATPase signaling in the production of excessive ROS contributes to the pro-oxidant state of cells, affecting cellular mechanisms. Further investigating the role of Na/K-ATPase signaling in perpetuating aging could provide potential therapeutic target for developing anti-aging intervention strategies. In this regard, the peptide, pNaKtide, has demonstrated profound effects as an antagonist of Na/K-ATPase signaling, contributing towards the inhibition of oxidant amplification loop and preventing disease progression. We provide a basis for new implications of Na/K-ATPase signaling in attenuating clinical disorders and suggest broader applications of this signaling cascade in reversing or preventing the development and progression of oxidative stress related pathologies.
